# Methylmalonic Acidemia: A Review of Cases in Jordan

**DOI:** 10.7759/cureus.94832

**Published:** 2025-10-17

**Authors:** Mo'men Alakil, Noor A Megdadi, Lina Alghonemeen, Sumaia Alrababah, Nahid Altawarah, Shahrazad Alqyam

**Affiliations:** 1 Department of Pediatrics, Jordanian Royal Medical Services, Amman, JOR

**Keywords:** acidurias, inborn error of metabolism, jordan, methylmalonic acidemia, organic acidemia

## Abstract

Background

This study reviews methylmalonic acidemias (MMAs), one of the organic acid disorders, to create a foundation for future studies by establishing a comprehensive database. Moreover, it contributes to a deeper awareness of the clinical features and outcomes observed in individuals affected by this condition.

Method

This case series includes all MMA diagnoses at the metabolic unit of Queen Rania Al Abdullah Hospital for Children in Amman, Jordan, from 2010 to 2023. The review included sociodemographic features, clinical and laboratory results, familial history, and parental consanguinity.

Results

Our cohort comprised 14 individuals (seven males, seven females) with a mean age at presentation of 12.9 months. Positive family history was observed in 35.7% of cases, and parental consanguinity was observed in 85.7%. Mortality was reported in two patients (14.3%) during the study period at 3.8 and four years of age. Encephalopathy was documented in 57.1%. Failure to thrive and developmental delay were reported in six (42.9%) and five (35.7%) patients, respectively. Hypoglycemia emerged as the most prevalent laboratory finding, affecting 71.4% of cases. Additionally, hearing loss was noted in four patients (28.6%).

Conclusions

Due to the difficulties and delays in diagnosing these organic acidemia disorders, we highly recommend implementing newborn screening as a critical step for early intervention and guidance. For early intervention and guidance, health care professionals should possess a thorough understanding of the clinical manifestations of these disorders and proficiency in interpreting results from biochemical tests.

## Introduction

Methylmalonic acidemias (MMAs) encompass a group of metabolic disorders characterized by the impaired conversion of methylmalonyl-CoA into succinyl-CoA. Propionyl-CoA, derived from the catabolism of various substances, is catalyzed by propionyl-CoA carboxylase to form D-methylmalonyl-CoA. Methylmalonyl-CoA epimerase subsequently converts it into L-methylmalonyl-CoA. Deficiency in methylmalonyl-CoA epimerase is a rare disorder leading to persistent elevations of propionate-related metabolites and methylmalonic acid [1,2]. While it may present with metabolic acidosis and ketosis, individuals with this deficiency generally appear more clinically stable than those with severe forms of MMA [3].

MMA exhibits a broad spectrum of severity, spanning from severely ill newborns to apparently asymptomatic adults. Severe cases can display symptoms such as lethargy, feeding difficulties, vomiting, sepsis-like manifestations, tachypnea, and hypotonia [4]. If left untreated, these symptoms can progress to hyperammonemic encephalopathy, coma, and potentially result in death [5]. Surviving infants may encounter recurrent acute metabolic episodes during catabolic states, leading to basal ganglia injury and subsequent movement disorders [6]. In between episodes, patients often exhibit hypotonia, feeding problems, and failure to thrive [7]. As individuals age, complications such as pancreatitis, bone marrow suppression, osteopenia, optic nerve atrophy, and renal issues may arise, occasionally necessitating renal transplants in severe cases [8]. Mild forms of the disorder may manifest later in life with symptoms such as hypotonia, failure to thrive, and developmental delay; however, neurocognitive development can remain normal [9]. It's worth noting that the episodic nature and biochemical features of MMA may be mistaken for ethylene glycol ingestion, as propionate peaks in blood samples can lead to misinterpretation in certain testing methods [10].

Laboratory findings include ketosis, metabolic acidosis, hyperglycinemia, hyperammonemia, hypoglycemia, anemia, neutropenia, thrombocytopenia, and the presence of significant quantities of methylmalonic acid in body fluids. Additionally, metabolites of propionic acid (3-hydroxypropionate and methylcitrate) are detected in the urine. The plasma acylcarnitine profile reveals elevated propionylcarnitine (C3) and methylmalonyl carnitine (C4DC). It is important to note that hyperammonemia in MMA may be mistaken for a urea cycle disorder [11].

The therapeutic approach to MMA involves supportive management during acute episodes and long-term strategies. Long-term treatment includes adherence to a dietary regimen based on recommended protein allowances and the oral administration of L-carnitine. Severe cases may necessitate adjustments in protein intake. Individuals with isolated MMA linked to cobalamin metabolism defects may respond positively to parenteral hydroxocobalamin [12,13]. Chronic acidosis is commonly addressed through bicarbonate replacement therapy, while hyperammonemia, though a persistent concern, infrequently requires extended treatment [14].

Timely management of triggers such as infection, fasting, trauma, surgery, and high-protein meals is crucial. Long-term challenges involve issues like poor appetite, protein over-restriction, and deficiencies in essential amino acids, often leading to early adoption of enteral feeding. Rigorous monitoring of parameters, including blood pH, amino acids, methylmalonate concentrations, growth indicators, kidney function, vision, hearing, and bone mineral density, is essential. Liver, kidney, or combined transplants offer partial relief from metabolic abnormalities, with kidney transplantation providing modest improvement in clinical stability but not addressing fundamental metabolic challenges [15,16].

This review aims to analyze cases of MMA in Jordan, establishing a comprehensive database to support future research. Additionally, it seeks to increase awareness of the clinical features and outcomes of affected individuals, thereby enhancing understanding of diagnostic challenges and informing improvements in future screening and management strategies.

## Materials and methods

In this retrospective study, we conducted a comprehensive analysis of patients diagnosed with MMA who were treated at the metabolic unit of the Queen Rania Al Abdullah Hospital for Children (QRHC). QRHC, a specialized pediatric referral center affiliated with King Hussein Medical City, is located in Amman, the capital of Jordan, and functions as a key center for diagnosing and treating complex metabolic disorders in the region. The study period spanned thirteen years, from 2010 to 2023, allowing for a broad evaluation of clinical patterns and outcomes across a substantial timeframe.

Approval for the study was obtained from the Royal Medical Services Human Research Ethics Committee (approval number: 12/2025-30). Patient information and clinical data were collected from two primary sources: the metabolic unit’s medical records and the hospital’s electronic archiving system. The dataset was designed to be comprehensive, incorporating demographic details, family medical histories, and the presence or absence of parental consanguinity. In addition, data on the initial clinical manifestations at the time of presentation, relevant biochemical and laboratory investigations, and follow-up outcomes were systematically recorded.

Biochemical diagnosis of MMA was confirmed by detecting elevated methylmalonate, with or without accompanying increases in 3-hydroxypropionate and 2-methylcitrate, on urine organic acid chromatography. Genetic confirmation was performed in cases showing elevated propionylcarnitine (C3) on acylcarnitine profiling but no detectable methylmalonate in urine, revealing homozygous pathogenic or likely pathogenic variants in the MMUT gene encoding methylmalonyl-CoA mutase. Patients with cobalamin-related MMA subtypes were excluded. This approach ensured a well-defined cohort and reliable assessment of the clinical spectrum, diagnostic challenges, and management patterns of MMA within the Jordanian healthcare setting.

## Results

This series included 14 patients, seven males and seven females. The mean age at presentation was 12.9 months (±12.1), and the mean age at the time of the study was 10.7 years (±6.3). Positive family history was identified in five patients (35.7%), and parental consanguinity was observed in 12 patients (85.7%). Mortality was reported in two patients (14.3%) during the study period at 3.8 and four years of age.

Table 1 illustrates the clinical manifestations of MMA. Encephalopathy was documented in eight patients (57.1%). Inadequate physical growth characterizes failure to thrive, whereas developmental delay involves delays in achieving milestones across various domains; these were reported in six (42.9%) and five (35.7%) patients, respectively. It is important to note that a child may experience developmental delay without concurrent failure to thrive and vice versa. Hypoglycemia emerged as the most prevalent laboratory finding, affecting 71.4% of cases. Additionally, hearing loss was noted in four patients (28.6%).

**Table 1:** Presentation of MMA (N = 14)

	N	%
Hyperammonemia	3	21.4
Hypoglycemia	10	71.4
Acidosis	3	21.4
Encephalopathy	8	57.1
Vomiting and refusing food	5	35.7
Hypotonia	5	35.7
Failure to thrive	6	42.9
Developmental delay	5	35.7
Hearing loss	4	28.6

Table 2 presents a case series involving 14 patients diagnosed with MMA. The evaluation of acylcarnitines is essential for the evaluation of metabolic acidosis. These substances are vital in transporting fatty acids and expelling organic acids. Supplementation of carnitine proves advantageous in eliminating metabolites in specific metabolic disorders. The naming convention for acylcarnitines depends on factors such as the number of carbons, double bonds, and hydroxyl groups.

**Table 2:** Case series of 14 patients with methylmalonic acidemia (MMA) *Passed during the study period C2: acetylcarnitine; C3: propionylcarnitine; C3-DC: malonylcarnitine; C3C2: high ratio of C3 compared to C2; C4-OH: 3-OH-iso-/butyrylcarnitine; C5-OH: 3-OH-isovalerylcarnitine; C6-DC: 3-methylglutarylcarnitine; C16: hexadecanoylcarnitine; F: female; M: male.; +: present; -: negative

Case number	Age at presentation	Age at study	Sex	Family history	Parental consanguinity	Acidosis	Hyperamonemia	Hypoglycemia	Encephalopathy	Vomiting	Hypotonia	Developmental delay	Hearing loss	Acylcarnitine
1	12	15	F	-	+	No	No	No	Yes	No	No	No	No	C3, C3C2, C3C16, C5OH, C4OH, C3DC, C6DC
2	7	14	M	+	+	No	No	No	No	No	No	No	No	C3, C3C2, C3C16
3	7	11.1	M	-	-	Yes	Yes	Yes	Yes	Yes	Yes	Yes	No	C3, C3C2, C3C16
4	18	13.2	F	+	+	No	No	Yes	Yes	No	No	No	No	C3, C3C2, C3C16
5	4	12	F	-	+	No	No	Yes	Yes	Yes	Yes	Yes	Yes	C3C2, C4OH
6	1	9.2	M	-	+	No	No	Yes	Yes	No	No	No	No	C3
7	36	19	M	-	+	No	No	Yes	No	No	No	No	No	C3, C3C2, C3C16
8	9	6	M	+	+	No	No	Yes	Yes	No	No	No	No	C3
9*	0.13	3.8	M	-	+	No	No	Yes	No	No	No	No	No	C3, C3C2, C3C16
10	24	21	F	+	+	No	No	Yes	No	No	No	No	No	C3, C3C2, C3C16
11	14	2.8	F	-	+	No	No	No	No	Yes	Yes	Yes	Yes	C3
12	0.07	1.7	F	+	+	No	Yes	Yes	Yes	Yes	Yes	Yes	Yes	C3, C3C2, C3C16
13	37	17	F	-	+	No	No	No	No	No	No	No	No	C3
14*	12	4	M	-	-	Yes	Yes	Yes	Yes	Yes	Yes	Yes	Yes	C3

## Discussion

QRHC is an integrated hospital located within King Hussein Medical City in Amman, the capital of Jordan. Serving as a specialized referral center for the entire kingdom. Due to the rarity of metabolic conditions and the need for specialized care, a majority of cases are directed to our units. As a result, studies conducted by the metabolic unit at QRHC are indicative of the metabolic disease landscape in Jordan.

While the exact epidemiology of metabolic diseases in Jordan has not been systematically investigated, the prevalence is expected to be higher due to elevated rates of consanguineous marriages. Consequently, it is imperative to undertake additional studies to comprehensively understand the prevalence of metabolic diseases, including MMA, in Jordan. This initiative aims to establish a robust foundation for subsequent inquiries, potentially leading to the formulation of a targeted screening program for metabolic diseases.

In a 2012 publication from our institution [17], a retrospective analysis was conducted on 51 patients diagnosed with organic acidemia who received treatment at a metabolic clinic within King Hussein Medical City over five years (2005-2010). Propionic acidemia emerged as the predominant subtype, constituting 27.5% of cases, followed by MMA at 11.8%. However, in twenty-nine patients, the specific subtype remained indeterminate. Nevertheless, this cohort manifested symptoms of acidotic respiration, with or without encephalopathy, accompanied by findings of wide anion gap metabolic acidosis, elevated ammonia, and lactate levels in blood gases.

Contrastingly, our clinic's records indicate 73 patients diagnosed with organic acidemia, wherein Isovaleric acidemia was the most prevalent subtype, accounting for 28.8% of organic acidemia cases. Subsequent in frequency was 3-hydroxy-3-methylglutaryl-CoA lyase deficiency, representing 20.5%, and MMA constituted the third most common disease with a frequency of 19.2%. Propionic acidemia occupied the fourth position in frequency, accounting for 16.4%. Notably, the disparity between recent and prior reviews could be attributed to the previous study's classification of 56.9% of organic acidemia subtypes as undiagnosed.

In a regional study from Syria [18], a country neighboring Jordan with similar population sociodemographic characteristics, seventy patients with organic acidemia were examined between 2008 and 2012, and MMA was identified as the most prevalent disorder (57.1%). In the Syrian study, a family history was identified in 42.5% of patients, compared to 35.7% in our review. Parental consanguineous marriages were observed in 77.5% of patients in the Syrian study, closely aligning with our findings of 85.7%. The mortality rate in the Syrian study was 17.5%, slightly higher than the 14.3% observed in our patients.

In another study from Lebanon [19] spanning 12 years (2008-2010), out of 83 patients diagnosed with organic acidemia, MMA was the most common type, accounting for 27.7%. Similarly, MMA was the most prevalent type in Tunisia, constituting 33.5% [20]; in Italy, it accounted for 24.4% [21].

Likely, some patients were not referred to our facility, possibly due to early mortality or seeking local treatment. Consequently, we acknowledge that the actual prevalence of MMA may exceed the estimates derived from our study. We advocate for including organic acidemia in national screening programs to enhance the well-being and care outcomes of individuals affected by these conditions.

A positive family history was identified in 35.7% of patients, and parental consanguinity was observed in 85.7%. These findings highlight the strong genetic contribution to MMA occurrence in Jordan. Despite advances in biochemical testing, limited access to molecular diagnostic facilities remains a major challenge. Establishing a national registry, expanding genetic testing services, and implementing genetic counseling programs could facilitate earlier diagnosis, improve prevention, and enhance healthcare planning for metabolic disorders in Jordan.

This study is limited by its small sample size, single-center retrospective design, incomplete follow-up, and lack of uniform genetic and biochemical data. Some patients may not have been referred to our center, potentially underestimating the true prevalence of MMA in Jordan. Despite these limitations, the study provides valuable insights into the clinical presentation and outcomes of MMA and lays the groundwork for future research and national screening initiatives.

## Conclusions

This case series delineates the clinical manifestations and outcomes of MMA in Jordan, highlighting the diagnostic challenges associated with delayed recognition and constrained healthcare resources. These findings reinforce the critical importance of implementing national newborn screening programs to facilitate early detection and timely intervention. Equally, strengthening physician awareness of the clinical and biochemical hallmarks of MMA is vital to ensure accurate diagnosis, effective management, and improved prognostic outcomes. Future multicenter and population-based investigations are warranted to more accurately determine the epidemiology of MMA and to inform evidence-based healthcare strategies for inherited metabolic disorders within the region.
